# Finger Flexor Force Influences Performance in Senior Male Air Pistol Olympic Shooting

**DOI:** 10.1371/journal.pone.0129862

**Published:** 2015-06-29

**Authors:** Daniel Mon, María S. Zakynthinaki, Carlos A. Cordente, Antonio J. Monroy Antón, Bárbara Rodríguez Rodríguez, David López Jiménez

**Affiliations:** 1 Universidad Politécnica de Madrid, Sports Department, Madrid, Spain; 2 Applied Mathematics and Computers Laboratory, Technical University of Crete, Crete, Greece; 3 Universidad Internacional de la Rioja, Physical Education, Madrid, Spain; 4 Universidad Francisco de Vitoria, Sports Department, Madrid, Spain; 5 Universidad Católica del Norte, Antofagasta, Chile; Universidad Europea de Madrid, SPAIN

## Abstract

The ability to stabilize the gun is crucial for performance in Olympic pistol shooting and is thought to be related to the shooters muscular strength. The present study examines the relation between performance and finger flexor force as well as shoulder abduction isometric force in senior male air pistol shooting. 46 Spanish national level shooters served as test subjects of the study. Two maximal force tests were carried out recording handgrip and deltoid force data under competition conditions, during the official training time at national Spanish championships. Performance was measured as the total score of 60 shots at competition. Linear regressions were calculated to examine the relations between performance and peak and average finger flexor forces, peak and average finger flexor forces relative to the BMI, peak and average shoulder abduction isometric forces, peak shoulder abduction isometric force relative to the BMI. The connection between performance and other variables such as age, weight, height, BMI, experience in years and training hours per week was also analyzed. Significant correlations were found between performance at competition and average and peak finger flexor forces. For the rest of the force variables no significant correlations were found. Significant correlations were also found between performance at competition and experience as well as training hours. No significant correlations were found between performance and age, weight, height or BMI. The study concludes that hand grip strength training programs are necessary for performance in air pistol shooting.

## Introduction

Olympic shooting is a high precision sport, where high level performance requires maximum control of all body movements. In air pistol the highest score refers to a very small area (diameter 11.5mm ± 0.1mm), compared to the long shooting distance of 10 m [1]. Nevertheless, elite pistol shooters are able to achieve scores as high as 594/600 (world record in males, 600 points being the maximum score), which is equivalent to a shooting accuracy of 99% [2].

Many are the factors that can influence performance in Olympic pistol shooting. A factor widely accepted to affect the movements of the gun and consequently performance is the shooter’s static balance [3]. The quantification, however, of the relation between static balance and performance is not clear in the existing literature.

The ability to stabilize the gun, related to movement of the various kinetic lines of the shooter’s body, also plays a very important role [4]. The movements of the centre of pressure (COP) on the X axis (mainly related to body movements) as well as on the Y axis (mainly related to shoulder and wrist movement control) have been associated to the vertical and the lateral movements of the gun respectively [4]. According to Tang, Zhang, Huang, Young, and Hwang [5], elite shooters minimize horizontal movements and are therefore able to better stabilize the gun, this way minimizing horizontal movements.

Although various authors [4], [5] report that part of the ability to stabilize the pistol is determined by the level of the shoulder and forearm muscular control, the number of studies that relate muscular factors or the physical condition of the shooter to performance is relatively low. Krasilshchikov, Zuraidee, and Singh [6] suggest that the implementation of a physical conditioning program together with technique training is essential for the improvement of the ability to stabilize the pistol. The study of Vercruyssen, Christina, & Muller [7] reports significant relations between shooting performance and both wrist strength (r = 0,82) and deltoid strength (r = 0,89).

According to Anderson and Plecas [8], the forearm muscle contraction peak force is significantly related with performance in pistol shooting (r = 0,38), however this particular study was unfortunately carried out on very heterogeneous groups of males and females. The same authors suggest that in order to be able to hold a 1kg pistol at a distance of 68.2 cm a muscular force of 7,2kg is required. The study of Yuan & Lee [9] supports this result, indicating in addition that changes in the length of the pistol or in the distribution of its weight affect its stability and consequently the performance of the shooter. Kennedy [10] reports that the strength, and in particular hand strength, has been recognized as a limiting factor in shooting accuracy, especially in females. On the other hand, the study of Mason, Cowan, & Gonczol [11], found no relation between the hand grip strength and shooting performance.

Books and journals specialized in Olympic shooting suggest that general physical condition programs [12], [13] as well as specific strength exercises [14] are necessary in order for performance to be improved. However, the study of Belinchon [15] reports that some factors regarding the physical condition of the Spanish Olympic shooters are inferior when compared to athletes of other sports. There is therefore the need for further study on the importance of physical conditioning in Olympic shooting.

It should be emphasized here that no studies were found in the literature that were carried out under competition conditions. It is a fact that all scientific background is based on data recorded under laboratory conditions or during training, which is a limiting factor for the validity of the conclusions of these studies, especially regarding further practical applications.

The objective of the present study is to examine the influence of various factors, mainly finger flexor forces and shoulder abduction isometric forces, but also factors related to training, or body morphology, on shooting performance in senior air pistol male shooters, under competition conditions.

## Materials and Methods

### Ethics statement

The Ethical Board of the Spanish Team Sports Association approved the experimental design of the study. The informed consent document that all the participants signed before data collection was also approved by the Ethical Board of the Spanish Team Sports Association. We confirm that our research meets the highest ethical standards for authors and co-authors. The study was performed following the guidelines of the Declaration of Helsinki, last modified in 2008.

The authors certify that the present research was carried out in the absence of any financial, personal or other relationships with other people or organizations that could inappropriately influence, or be perceived to influence, the presented work and lead to a potential conflict of interest.

### Participants

46 male senior pistol shooters who competed in the air pistol Spanish Championship in December 2012 participated in the present study (the senior category is referred to ages between 21 and 54 years old [1]. The participants’ characteristics are shown in Table 1.

**Table 1:** Mean (± s) values of the participants’ characteristics.

Age (years)	42,70 ± 10,67
Height (m)	1,75 ± 0,07
Weight (Kg)	87,38 ± 13,30
BMI (kg/m^2^)	28,60 ± 4,60
Experience (years)	11,92 ± 8,90
Training (hours/week)	5,70 ± 5,64

The participation in the study was voluntary and open to all competing shooters. According to the regulations of the Spanish Federation of Olympic Shooting eligibility to compete required a minimum score of 510 points in men air pistol in previous national competitions [1].

### Apparatus-equipment

The participants’ performance was measured by use of official paper targets, according to the Spanish Shooting Federation Rules and Regulations [1] and as provided by the referees of the Spanish Olympic shooting federation after the competition. A handheld dynamometer Takei A5401 was used to measure the finger flexor force. Shoulder abduction isometric forces were measured by use of a 16 kg weight attached to a Kistler force platform 9286AA by means of a rigid steel wire. The height of the measurement system was adjustable to allow measurements for all participants.

### Experimental protocol

The protocol consisted of two tests:

1. During the first test the hand dynamometer was used to measure the participants finger flexor force. The measurements were repeated three times with the arm normally used by the shooters to shoot extended along the body. The shooters were asked to perform a maximal contraction of the flexors of the fingers without bending the elbow.
2. During the second test a maximal isometric contraction of deltoid abduction was performed, starting from an initial position of 90° shoulder abduction. The measurements were repeated three times. To calculate the force exerted by the athlete, the minimum weight measured by the platform during the test was subtracted from the 16 kg of the weight which was placed on it.

Performance was measured during 60 shots under competition conditions in the “air pistol Spanish Championship”, in December 2012, in Madrid. Taking into account the characteristic of such a maximal force test [16], a minimum rest of 60 seconds was allowed between measurements for both tests.

Both tests were carried out on the day previous to that competition and during the official training time.

### Statistical analysis

The Kolmogorov-Smirnov test was used to determine the goodness of fit to the normal distribution of the variables. To examine the relations between performance, hand flexor peak force and peak isometric force of the deltoids of the participants, simple linear regression was used. The level of significance was set to 0.05. The statistical analysis of the variables was performed using SPSS PASW Statistics 17.

### Variables

For the purposes of the present study the following variables were analysed, (please refer to Table 2): Regarding the profile of the participants: Age, weight, height, BMI, experience in years, training hours per week and performance (mean value over 60 shots); regarding the strength of the participants: Peak finger flexor force, mean finger flexor force, peak finger flexor force relative to the BMI, peak isometric shoulder abduction force, mean isometric shoulder abduction force, peak isometric shoulder abduction force relative to the BMI.

**Table 2:** Mean (± s) of the recorded data. N = 46.

Performance (points over 60 shots)	548,22 ± 13,70
Mean finger flexor force (Kg)	47,92 ± 7,26
Peak finger flexor force (Kg)	49,96 ± 7,24
Peak finger flexor force relative to BMI	173,90 ± 42,77
Mean isometric force, shoulder abduction (Kg)	9,95 ± 2,03
Peak isometric force, shoulder abduction (Kg)	10,33 ± 2,05
Peak isometric force, shoulder abduction relative to BMI	35,83 ± 9,43

## Results

### Correlations regarding performance

Significant correlations were found between performance at competition and experience (*F*_*1*,*43*_ = 7.18, *p*<0.05), training hours (*F*_*1*,*43*_ = 11.51; *p*<0.01), mean finger flexor force (*F*_*1*,*43*_ = 6.63; *p*<0.05) and peak finger flexor force (*F*_*1*,*43*_ = 6.02; *p*<0.05). For the rest of the force variables no significant correlations were found (Table 3).

**Table 3:** Linear regression between performance and *exp* (experience), *trn* (training), *Ffm* (mean finger flexor force), *Ffp* (peak finger flexor force), *Fsm* (mean isometric shoulder abduction force), *Fsp* (peak isometric shoulder abduction force), *Ffrel* (peak finger flexor force relative to the BMI) and *Fsrel* (peak isometric shoulder abduction force relative to the BMI).

	*r*	*r* ^*2*^ corrected	CV% = SD/M x 100
*exp*	0,38	0,12	2,34**
*trn*	0,46	0,19	2,24***
*Ffm*	0,37	0,11	2,35**
*Ffp*	0,35	0,1	2,37**
*Fsm*	0,25	0,04	2,46*
*Fsp*	0,29	0,06	2,43*
*Ffrel*	0,25	0,04	2,45*
*Fsrel*	0,27	0,05	2,43*

*** significance level 0.01.

**significance level 0.05.

* significance level >0.05.

No significant correlations were found between performance and age, weight, height or BMI (*p*>0.05) (Table 3).

## Discussion and Conclusion

The novelty and importance of the present study lies on the fact that it is based on data recorded under actual competition conditions and not in a laboratory, associating performance with the actual competition results. This way the conclusions of this study can provide coaches and scientist with a clearer idea of the importance of complementary strength training programs.

While many are the factors that affect performance in Olympic shooting, the majority of the studies agree that the ability to stabilize the gun is crucial [3]. Certain movements of the gun, strongly related to the kinetics of the body [4], are associated to the muscular abilities of the wrist and the shoulder [4], [5].

Although the exact quantification of the influence of physical conditioning and muscular strength on pistol shooting performance is not possible, the results of the present study suggest that in senior male air pistol Olympic shooting the finger flexor force influence performance. In contrast to the results presented in [11], a coefficient of variation between CV = 2.35–3.37 was found for performance related to the force exerted by the forearm. However, no influence was found between the force exerted by the shoulder and the performance.

In agreement with the results presented by Anderson and Plecas [8], who found a *r* ^*2*^ = 0.14 correlation between performance and finger flexor force, we found a similar correlation of *r* ^*2*^ = 0.11. Also, in agreement with the results of Vercruyssen et al. [7], we found significant correlations between performance and finger muscular force. Our results do not coincide, however, regarding the quantification of these correlations (these authors report much higher Pearson correlation values). In contrast to Vercruyssen et al. [7], we found no significant correlation between performance and shoulder abduction force, however, a positive linear tendency was found, requiring further analysis in future studies.

In accordance to Belinchon [15], no significant correlation was found between performance and body variables. This result supports the conclusion of Belinchon [15] that in shooting no preference biotype seems to exist.

The relatively low correlations obtained, ranging between r^2^(corrected) = 0.1 and r^2^(corrected) = 0.19, should be considered important, taking into account the high precision (99%) required for success in high level pistol shooting such that even decimal points are crucial for the final result of the competition [2]. To really understand the importance of our results, one should take into account that a CV between 2.35–2.37 is equivalent to 14 points (maximum 600) at competition. In high level shooting such a difference is very important as an analysis of the world championships from 1998 to nowadays show that this 14 points is equivalent to an average of 57 positions in the final ranking [17].

In agreement with existing books and journals specialized in Olympic shooting the findings of the present study suggest that specific strength exercises [14] are necessary to improve performance. We therefore conclude that training programs for hand grip strength should be necessary in pistol shooting.

This paper can be served as a base for future studies that will include different types of muscular strength and apply the analysis to different population groups or different shooting modalities.
